# Zinc finger protein 468 up-regulation of TFAM contributes to the malignant growth and cisplatin resistance of breast cancer cells

**DOI:** 10.1186/s13008-024-00113-1

**Published:** 2024-03-01

**Authors:** Zhaoyang Jia, Feng Wang, Gongzhuo Li, Ping Jiang, Yuanxiu Leng, Longzhu Ke, Li Luo, Wei Gao

**Affiliations:** 1 Department of Radiation Oncology, Shanghai Fourth People’s Hospital, School of Medicine, Tongji University, Shanghai, China; 2 Department of Oncology, GuiHang Guiyang Hospital, Guiyang, China; 3 Department of Radiation Oncology, Shanghai Tenth People’s Hospital, Tongji University School of Medicine, Shanghai, China; 4 Hubei University of Chinese Medicine, Wuhan, China

**Keywords:** Breast cancer, ZNF468, TFAM, Cisplatin

## Abstract

**Background:**

Because of the progress on the diagnosis and treatment for patients with breast cancer (BC), the overall survival of the patients has been improved. However, a number of BC patients cannot benefit from the existing therapeutic strategies as the essential molecular events triggering the development of BC are not well understood. Previous studies have shown that abnormal expression of zinc finger proteins is involved in the development of various malignancies, whereas it remains largely unclear on their significance during the progression of BC. In this study, we aimed to explore the clinical relevance, cellular function and underlying mechanisms of zinc finger protein 468 (ZNF468) in BC.

**Methods:**

The clinical relevance of ZNF468 and TFAM was analyzed based on TCGA database. Overexpression or knockdown of ZNF468 and TFAM were performed by transfecting the cells with overexpression plasmids and siRNAs, respectively. Overexpression and knockdown efficacy was checked by immunoblotting. CCK-8, colony formation, transwell and apoptosis experiments were conducted to check the cellular function of ZNF468 and TFAM. The content of mtDNA was measured by the indicated assay kit. The effects of cisplatin on BC cells were detected by CCK-8 and colony formation assays. The regulation of ZNF468 on TFAM was analyzed by RT-qPCR, immunoblotting, dual luciferase activity and ChIP-qPCR assays.

**Results:**

ZNF468 was overexpressed in BC patients and inversely correlated with their prognosis. Based on overexpression and knockdown assays, we found that ectopic expression of ZNF468 was essential for the proliferation, growth and migration of BC cells. The expression of ZNF468 also negatively regulated the sensitivity of BC cells to the treatment of cisplatin. Mechanistically, ZNF468 potentiated the transcription activity of TFAM gene via direct binding on its promoter. Lastly, we demonstrated that ZNF468 up-regulation of TFAM was important for the growth, migration and cisplatin resistance in BC cells.

**Conclusion:**

Our study indicates that ZNF468 promotes BC cell growth and migration via transcriptional activation of TFAM. ZNF468/TFAM axis can serve as the diagnostic and therapeutic target, as well as the predictor of cisplatin effectiveness in BC patients.

**Supplementary Information:**

The online version contains supplementary material available at 10.1186/s13008-024-00113-1.

## Background

Breast cancer (BC) is one of the most common malignant tumors threatening women's health in the world, with the incidence ranking first and the mortality ranking second in female malignant tumors [1]. Based on the expression of the estrogen receptor (ER), progesterone receptor (PR), or epidermal growth factor 2 (HER2), BC patients are divided into three types, including ER+/HER2−, HER+ and ER−/PR−/HER2− type. ER+/HER2− type accounts for 70% of all the cases, while the remaining 15% cases are HER2+and ER−/PR−/HER2− (triple-negative breast cancer, TNBC), respectively [2]. Recently, increasing evidences have identified the critical molecular events driving the development of BC. For example, poly (ADP-ribose) polymerase (PARP), epidermal growth factor receptor (EGFR) and vascular endothelial growth factor (VEGF) are important drivers during the progression of TNBC and BRCA1- or BRCA2-mutated BC [3]. Benefiting from these studies, the inhibitors of PARP1 were under clinical evaluation for the treatment of BC patients [4–6]. Despite these progresses, a large number of TNBC patients are left without effective medication. Thus, it is urgent for us to identify novel driver genes which can be applied as the diagnostic or therapeutic target of BC patients.

The family of zinc finger proteins (ZNFs) comprises of thousands of proteins, exhibiting either transcription or post-transcription activity based on their interaction with DNA, RNA or proteins [7]. Previous studies have shown that ZNFs play important roles during the progression of cancers. For example, ZNF306 is amplified and overexpressed in colorectal cancer (CRC) patients. Overexpression of ZNF306 drives the tumorigenesis and 5-fluorouracil resistance of CRC through transcriptional activation of integrin β4 and VEGF [8]. Another study reported that ZNF306 functioned as an oncogene in multiple myeloma by modulating the expression of Cyclin D2 [9]. Similar with ZNF306, ZNF468 protein has C2H2-type zinc finger motif and is predicted to enable DNA-binding transcription factor activity by interacting with RNA polymerase II. However, the significance of ZNF468 in the development of cancers, including BC, remains to be understood.

In this study, we explored the clinical significance, cellular function and molecular mechanisms of ZNF468 in breast cancer cell growth and cisplatin sensitivity based on TCGA database, overexpression and knockdown of ZNF468, as well as CCK-8, colony formation, Transwell, luciferase activity and ChIP-qPCR assays. We demonstrated that ZNF468 up-regulation of TFAM not only contributed to the growth and migration of breast cancer cells but also reduced the sensitivity of the cells to cisplatin treatment.

## Results

### ZNF468 is overexpressed in BC patients

**Fig. 1:**
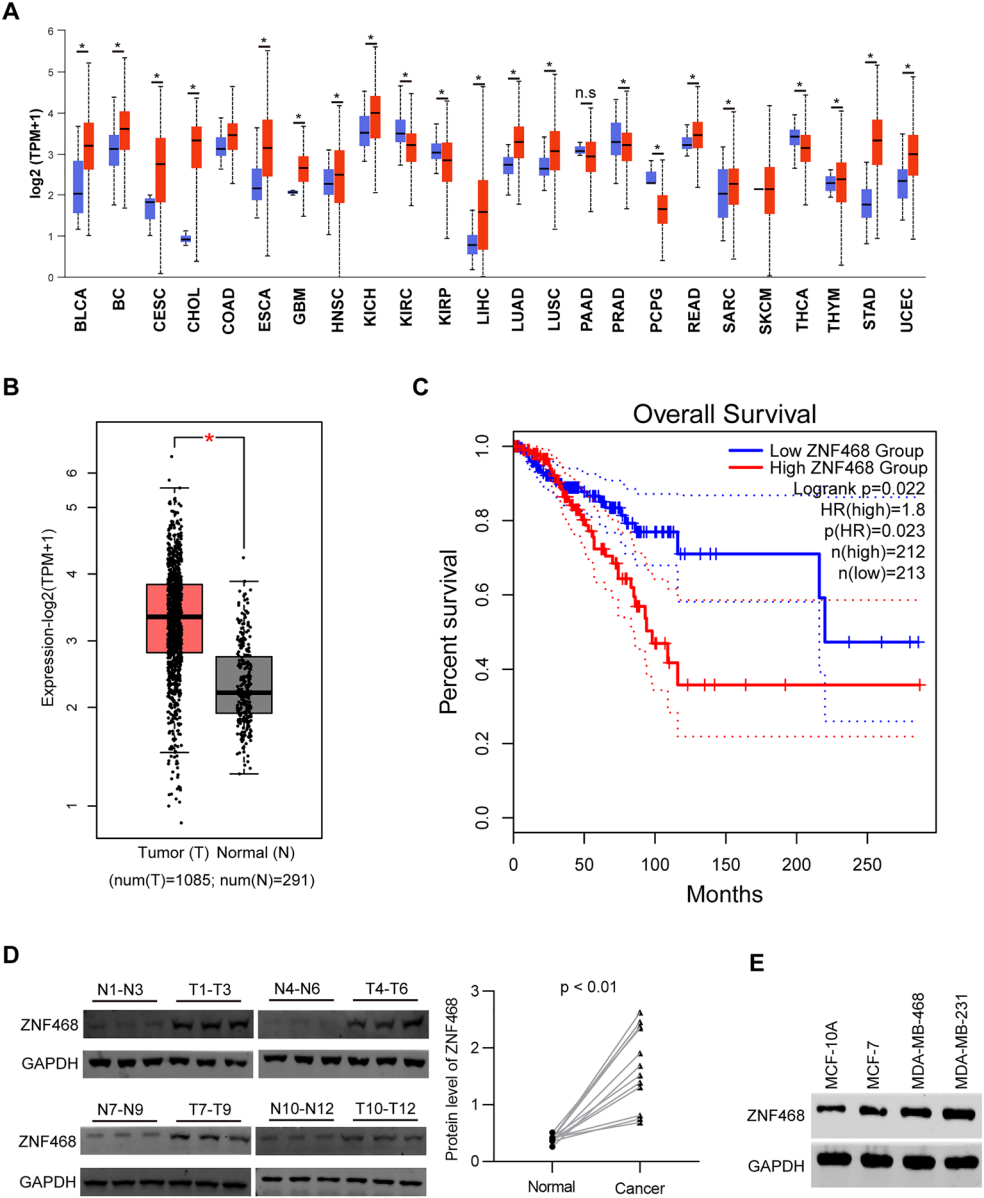
BC samples and cells have higher expression of ZNF468. **A** The transcript level of ZNF468 was analyzed in pan-cancer based on the database from TCGA. Most of the cancers showed higher expression ZNF468. n.s, no significance. *p < 0.05. **B** Analysis of ZNF468 transcript was conducted in cancer (n = 1085) and normal tissues (n = 291) derived from BC patients. p < 0.05. **C** Overall survival was analyzed on BC patients who were divided into ZNF468 high expression (n = 212) and low expression (n = 213) group. p = 0.022. **D** Cancer (n = 12) and normal tissues (n = 12) of BC patients were collected for immunoblotting analysis of ZNF468. Quantification of ZNF468 protein abundance was adjusted to GAPDH. p < 0.01. **E** The expression of ZNF468 was determined by immunoblotting assay in breast immortalized cells MCF-10A and in BC cells MCF-7, MDA-MB-468 and MDA-MB-231. For immunoblotting assays, the blots were cut prior to hybridisation with antibodies

Firstly, we investigated the clinical relevance of ZNF468 in pan-cancer. Based on TCGA database, ZNF468 was overexpressed in different cancer types, including bladder cancer (BLCA), Cervical squamous cell carcinoma and endocervical adenocarcinoma (CESC), glioma (GBM), lung adenocarcinoma (LUAD), breast cancer (BC) and other cancer types (Fig. 1A, B). We focused on BC and further found that LumA and LumB patients had higher expression of ZNF468 than HER+ and TNBC patients (Additional file 1: Fig. S1A). In addition, BC patients with high expression of ZNF468 had significantly poorer survival than those with low expression of ZNF468 (Fig. 1C). High expression of ZNF468 was significantly correlated with the poor survival of LumA patients, but not other subtypes (Additional file 1: Fig. S1B–E). To verify the results in Chinese patients, we collected the normal and cancer samples from BC patients and subjected them to immunoblotting analysis of ZNF468. The results showed that ZNF468 was highly expressed in the cancer tissues than normal tissues (Fig. 1D). Lastly, the expression of ZNF468 was also increased in BC cancer cell lines as compared with breast immortalized cells MCF-10A (Fig. 1E). Collectively, ZNF468 likely functions as an oncogene in BC.

### ZNF468 overexpression is essential for BC cell growth and migration

**Fig. 2:**
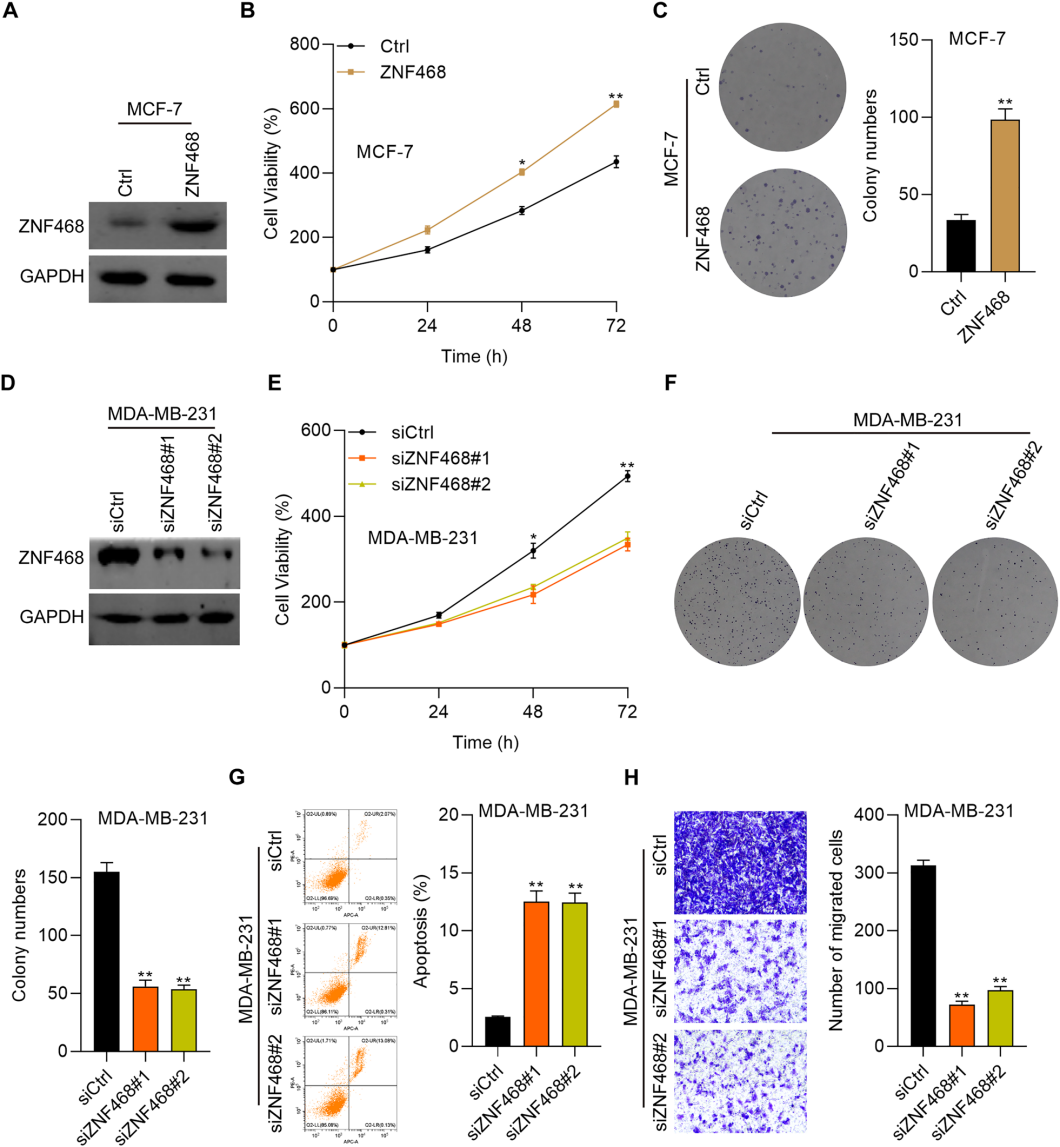
ZNF468 promotes the growth and migration of BC cells. **A** Immunoblotting analysis of ZNF468 in MCF-7 cells transfected with empty control and ZNF468 overexpression plasmids. GAPDH was used as internal control. **B** Cell viability was measured by CCK-8 assay for MCF-7 cells transfected with empty control and ZNF468 overexpression plasmids. *p < 0.05. **p < 0.01. **C** Colony formation assay was performed for Ctrl and ZNF468 overexpressing MCF-7 cells. **p < 0.01. **D** Immunoblotting detection of ZNF468 in MDA-MB-231 cells transfected with siCtrl, siZNF468#1 and siZNF468#2. GAPDH was used as internal control. **E**, **F** CCK-8 (**E**) and colony formation (**F**) assay was performed to analyze the cell proliferation of siCtrl, siZNF468#1 and siZNF468#2 MDA-MB-231 cells. *p < 0.05. **p < 0.01. **G** Cell apoptosis was checked by Annexin V/PI staining on flow cytometry system for siCtrl, siZNF468#1 and siZNF468#2 MDA-MB-231 cells. **p < 0.01. **H** Cell migration was checked by Transwell asasy for siCtrl, siZNF468#1 and siZNF468#2 MDA-MB-231 cells. **p < 0.01. For immunoblotting assays, the blots were cut prior to hybridisation with antibodies

Next, we intended to determine the cellular function of ZNF468 based on overexpression and knockdown experiments. Because MDA-MB-231 cells had relatively higher expression of ZNF468 than MCF-7 cells, we performed knockdown assay in MDA-MB-231 cells and overexpression assay in MCF-7 cells. Western blot demonstrated that ZNF468 was successfully overexpressed in MCF-7 cells by transfecting with overexpression vectors of ZNF468 (Fig. 2A). CCK-8 and colony formation results showed that ectopic expression of ZNF468 promoted the proliferation and growth ability of MCF-7 cells (Fig. 2B, C). Furthermore, we transfected MDA-MB-231 cells with siRNAs to knock down ZNF468. Immunoblotting results confirmed the knockdown efficacy (Fig. 2D). As expected, ZNF468 down-regulation suppressed the viability of MDA-MB-231 cells (Fig. 2E, F). The apoptosis of MDA-MB-231 cells was obviously induced by knockdown of ZNF468 (Fig. 2G). In addition, ZNF468 knockdown also inhibited the migration ability of MDA-MB-231 cells (Fig. 2H). These results suggest that ZNF468 acts as an oncogenic protein in BC by promoting the proliferation and migration capacity of BC cells.

### ZNF468 expression dictates the sensitivity of BC cells to cisplatin treatment

**Fig. 3:**
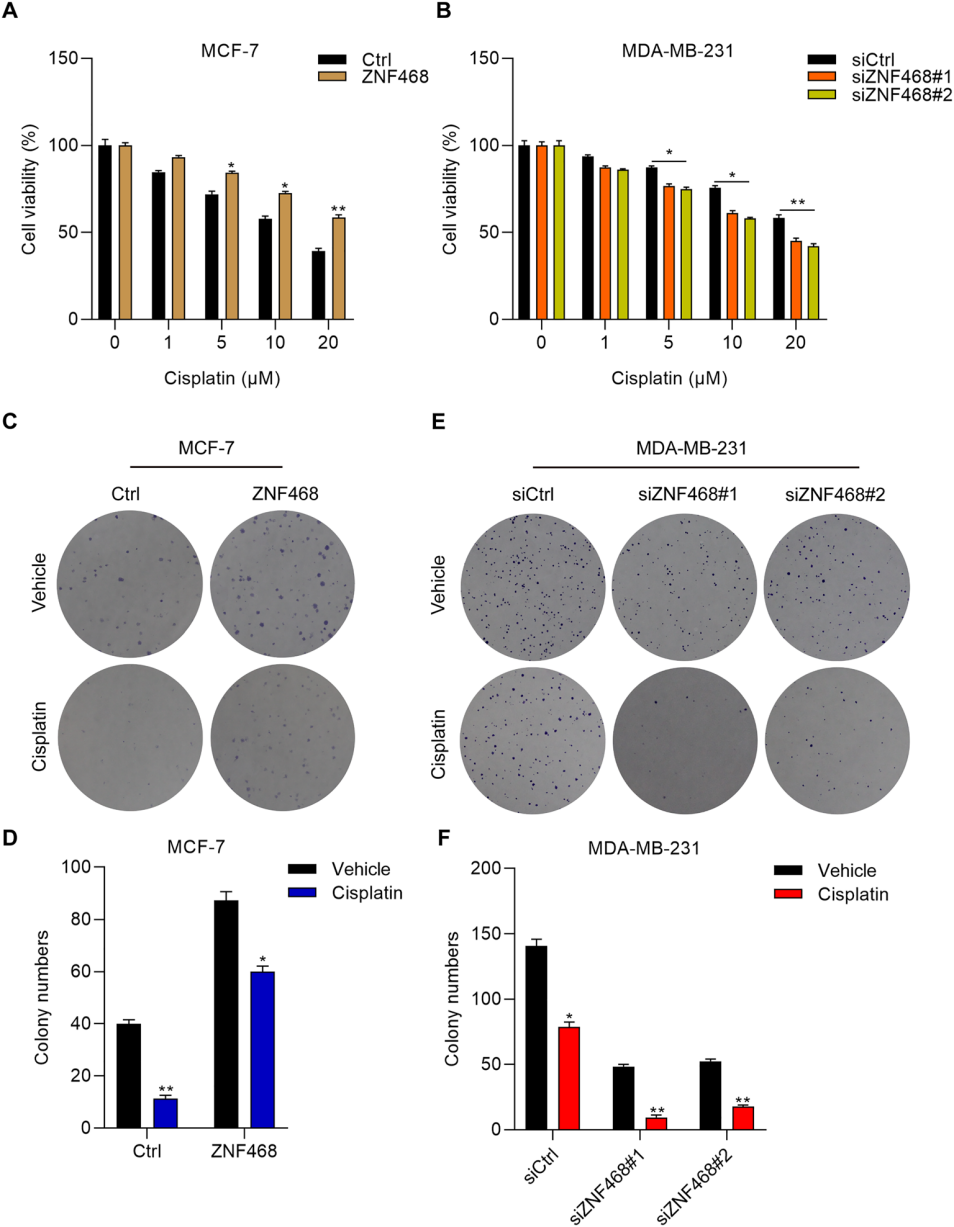
The abundance of ZNF468 confers the cytotoxicity of cisplatin to BC cells. **A** Ctrl and ZNF468 overexpressing MCF-7 cells were incubated with vehicle or different concentrations (1, 5, 10 and 20 μM) of cisplatin for 48 h. Cell viability was detected by CCK-8 assay. *p < 0.05. **p < 0.01. **B** siCtrl, siZNF468#1 and siZNF468#2 MDA-MB-231 cells were treated with vehicle or different concentrations (1, 5, 10 and 20 μM) of cisplatin for 48 h. Cell viability was detected by CCK-8 assay. *p < 0.05. **p < 0.01. **C**, **D** Ctrl and ZNF468 overexpressing MCF-7 cells were incubated with vehicle or 10 μM of cisplatin. Conoly formation was detected 10 days later. *p < 0.05. **p < 0.01. **E**, **F** siCtrl, siZNF468#1 and siZNF468#2 MDA-MB-231 cells were incubated with vehicle or 10 μM of cisplatin. Conoly formation was detected 7 days later. *p < 0.05. **p < 0.01

Cisplatin is a chemotherapeutic drug applied for the treatment of patients with advanced HER+ and TNBC breast cancer in clinic [10, 11]. To investigate whether ZNF468 dysregulation modulates the effectiveness of cisplatin, the BC cells transfected with ZNF468 siRNAs or overexpression vectors were treated with different concentrations of cisplatin and subjected to CCK-8 analysis of cell viability. As shown in Fig. 3A, when cisplatin inhibits the viability of MCF-7 cells transfected with empty control vectors at a dosage dependent manner, it exhibited lower inhibitory effects on the cells transfected with ZNF468 overexpression vectors (Fig. 3A). By contrast, ZNF468 knockdown enhanced the cytotoxicity of cisplatin on MDA-MB-231 cells, especially at higher concentration (Fig. 3B). These results indicated that ZNF468 overexpression protected MCF-7 cells from the toxicity of cisplatin, while ZNF468 knockdown promoted the inhibitory effect of cisplatin to MDA-MB-231 cells. To confirm the results, BC cells treated with cisplatin were subjected to colony formation experiments. As 10 μM of cisplatin had nearly 50% and 30% inhibitory effect on the proliferation of Ctrl MCF-7 and siCtrl MDA-MB-231 cells for 48 h, we applied this dosage in colony formation assay. We found that cisplatin exhibited higher cytotoxicity on the colonies of MCF-7 cells transfected with empty control vectors as compared with those with ZNF468 overexpression (Fig. 3C, D). In addition, knockdown of ZNF468 significantly potentiated the inhibitory effect of cisplatin on the colony formation ability of MDA-MB-231 cells (Fig. 3E, F). These results indicate that ZNF468 expression confers the sensitivity of BC cells to cisplatin treatment.

### ZNF468 potentiates the transcription activity of TFAM gene

**Fig. 4:**
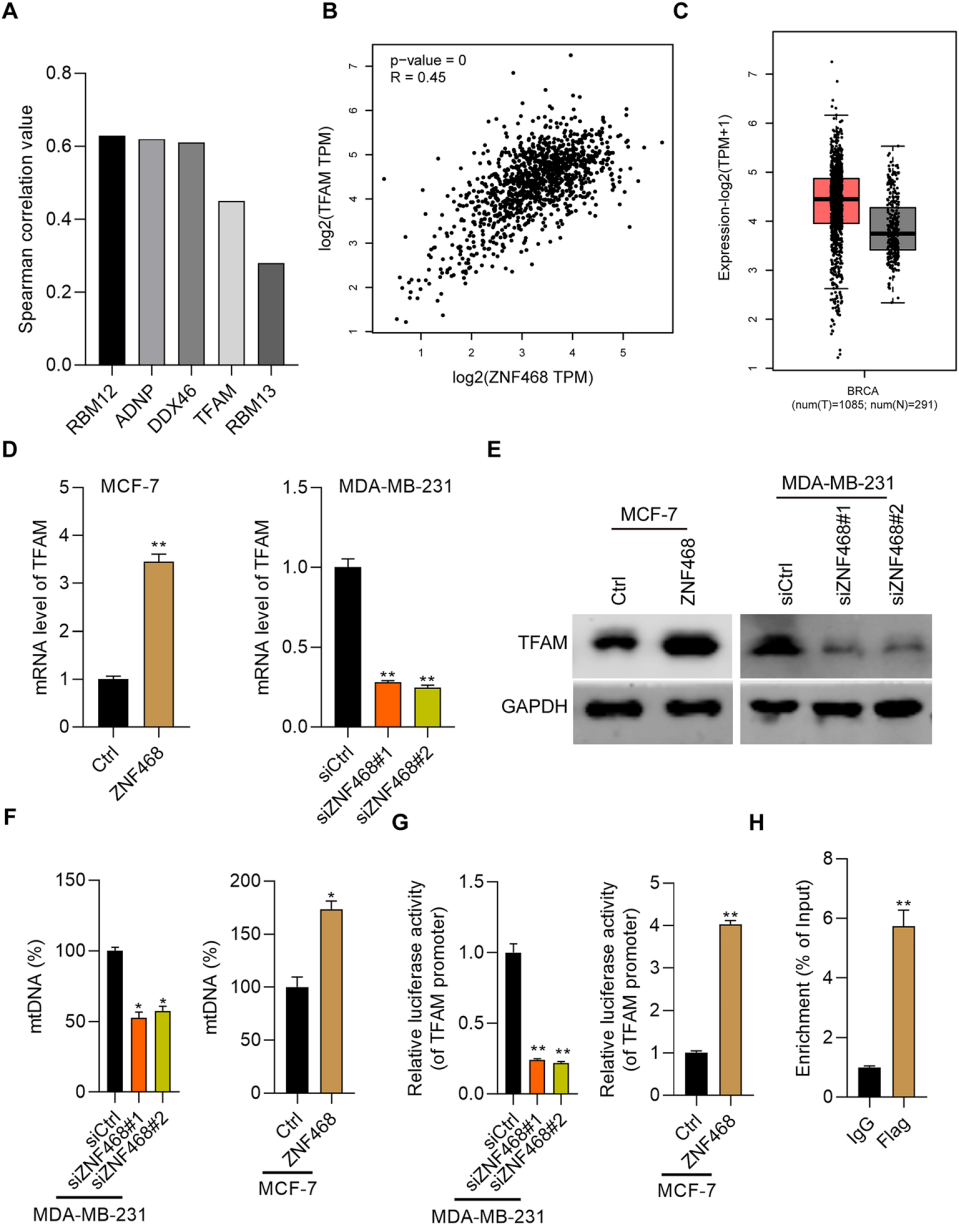
ZNF468 potentiates the expression of TFAM at transcriptional and protein level. **A** The positive genes correlated with ZNF468 in BC patients were analyzed based on TCGA database. **B** Spearman correlation between ZNF468 and TFAM was analyzed in BC patients from TCGA database. p < 0.001. **C** Analysis of TFAM transcript was performed in cancer (n = 1085) and normal tissues (n = 291) derived from BC patients. **D**, **E** The mRNA (**D**) and protein (**E**) level of TFAM was detected by RT-qPCR in Ctrl and ZNF468 overexpressing MCF-7 cells, and in siCtrl, siZNF468#1 and siZNF468#2 MDA-MB-231 cells. **p < 0.01. **F** Relative mtDNA copy number was measured by RT-qPCR assay in Ctrl and ZNF468 overexpressing MCF-7 cells, and in siCtrl, siZNF468#1 and siZNF468#2 MDA-MB-231 cells. *p < 0.05. **G** Dual luciferase activity of TFAM promoter was checked in Ctrl and ZNF468 overexpressing MCF-7 cells, and in siCtrl, siZNF468#1 and siZNF468#2 MDA-MB-231 cells. **p < 0.01. **H** ChIP-qPCR assay was performed to determine the binding of ZNF468 on the promoter of TFAM gene in MCF-7 cells. **p < 0.01. For immunoblotting assays, the blots were cut prior to hybridisation with antibodies

Because ZNF468 is a transcription factor, the downstream effectors participating in its function on BC growth and cisplatin should be illustrated. We analyzed the positively correlated genes of ZNF468 in BC patients based on TCGA database and found that RBM12, ADNP, DDX46, TFAM and RBM13 were positively correlated with ZNF468 (Fig. 4A). Previous studies demonstrated that down-regulation of TFAM was associated with the enhanced sensitivity of BC cells to cisplatin treatment [12]. Furthermore, we found that knockdown of TFAM not only suppressed the growth and proliferation of BC cells, but also promoted to toxicity of cisplatin in vitro and in vivo [13]. Thus, we intended to explore whether ZNF468 supported BC growth and cisplatin resistance through regulating TFAM. According to TCGA data, TFAM was positively correlated with ZNF468 and it was highly expressed in BC patients (Fig. 4B, C). RT-qPCR and immunoblotting assays showed that ZNF468 overexpression promoted, while its down-regulation suppressed the expression of TFAM at both mRNA and protein levels (Fig. 4D, E). We also checked the mtDNA content and found that ZNF468 positively regulated the levels of mtDNA in BC cells (Fig. 4F). Luciferase reporter results found that ZNF468 positively regulated the transcription activity of TFAM gene (Fig. 4G). Importantly, ZNF468 directly interacted with the promoter sequence of TFAM gene (Fig. 4G). These results indicate that ZNF468 transcriptionally activates TFAM gene in BC cells.

### ZNF468 contributes to the growth, migration and cisplatin resistance of BC cells through up-regulation of TFAM

**Fig. 5:**
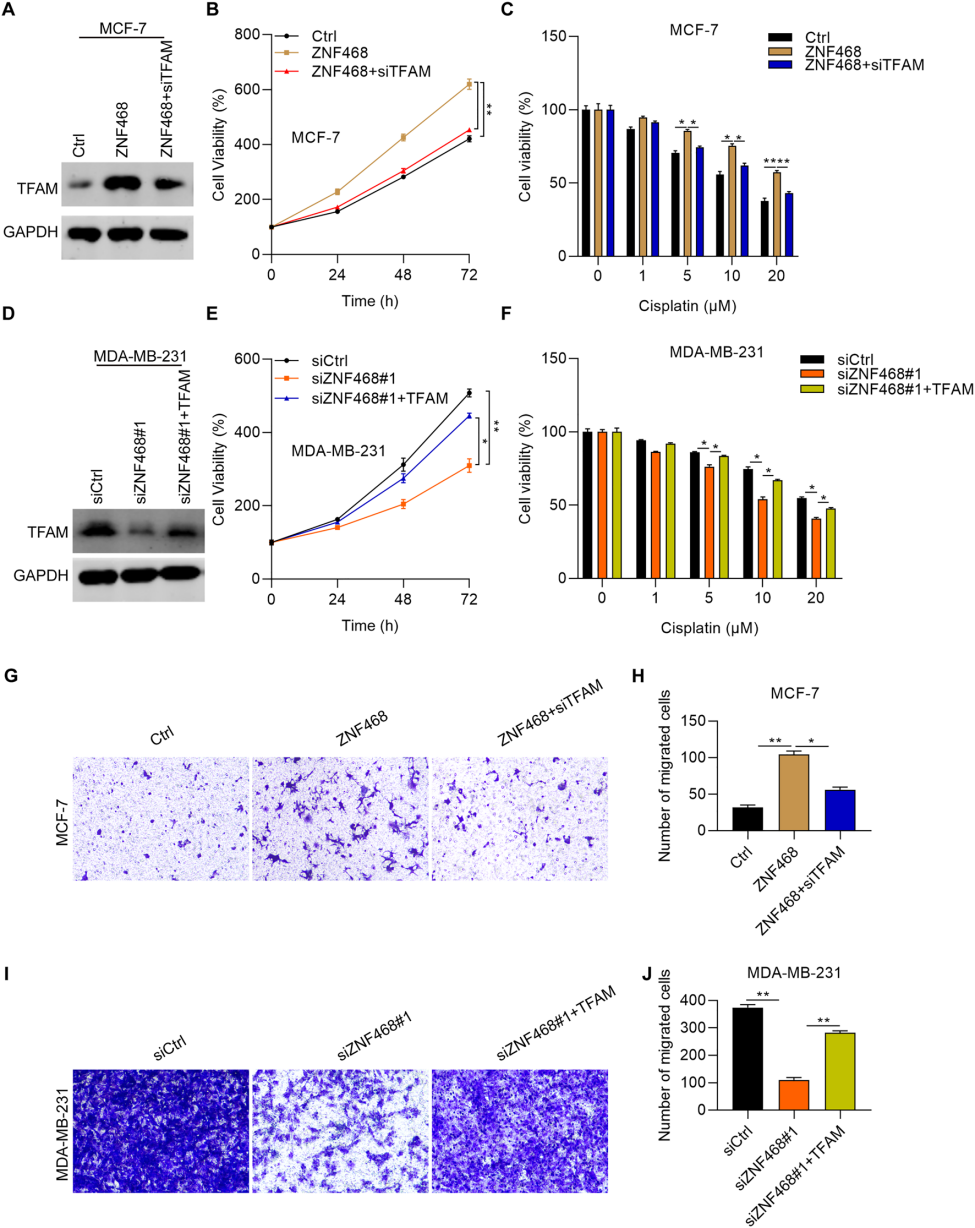
ZNF468 promotes the growth, migration and cisplatin resistance of BC cells through up-regulation of TFAM. **A** Immunoblotting assay was performed to check the protein expression of TFAM in Ctrl, ZNF468 and ZNF468+siTFAM MCF-7 cells. **B** CCK-8 was conducted to detect the viability of MCF-7 cells as described in A. **p < 0.01. **C** MCF-7 cells described in A were treated with vehicle and different concentrations of cisplatin (1, 5, 10 and 20 μM) for 48 h. Cell viability was detected by CCK-8 assay. *p < 0.05. **p < 0.01. **D** Immunoblotting assay was performed to check the protein expression of TFAM in siCtrl, siZNF468#1 and siZNF468#1+TFAM MDA-MB-231 cells. **E** CCK-8 was conducted to detect the viability of MDA-MB-231 cells as described in D. *p < 0.05. **p < 0.01. **F** MDA-MB-231 cells as described in D were treated with vehicle and different concentrations of cisplatin (1, 5, 10 and 20 μM) for 48 h. Cell viability was detected by CCK-8 assay. *p < 0.05. **G**, **H** Cell migration was checked by Transwell asasy for Ctrl, ZNF468 and ZNF468+siTFAM MCF-7 cells. *p < 0.05. **p < 0.01. **I**, **J** Cell migration was checked by Transwell asasy for siCtrl, siZNF468#1 and siZNF468#1+TFAM MDA-MB-231 cells. **p < 0.01. For immunoblotting assays, the blots were cut prior to hybridisation with antibodies

Lastly, we performed experiments to investigate the involvement of TFAM in ZNF468 triggering BC growth and cisplatin treatment. TFAM was knocked down in ZNF468 overexpressing MCF-7 cells and was ectopically expressed in ZNF468 silencing MDA-MB-231 cells. The cells were treated with or without different concentrations of cisplatin and then subjected to CCK-8 detection of cell viability. We found that TFAM knockdown largely reversed the enhanced cell proliferation of MCF-7 cells by ZNF468 overexpression (Fig. 5A, B). Interestingly, the reduced sensitivity of MCF-7 cells to cisplatin caused by ZNF468 was restored by knockdown of TFAM (Fig. 5C). By contrast, TFAM overexpression had promoting function not only on the proliferation but also on cisplatin resistance in ZNF468 silenced MDA-MB-231 cells (Fig. 5D–F). We also assessed the significance of ZNF468/TFAM axis on BC cell migration and found that knockdown of TFAM significantly reduced the migration ability of MCF-7 cells which was potentiated by overexpression of ZNF468 (Fig. 5G, H). Opposite results were shown after overexpressing TFAM in ZNF468 silenced MDA-MB-231 cells (Fig. 5I, J). These results suggest that ZNF468 promotes BC cell growth, migration and cisplatin resistance through up-regulation of TFAM.

**Fig. 6:**
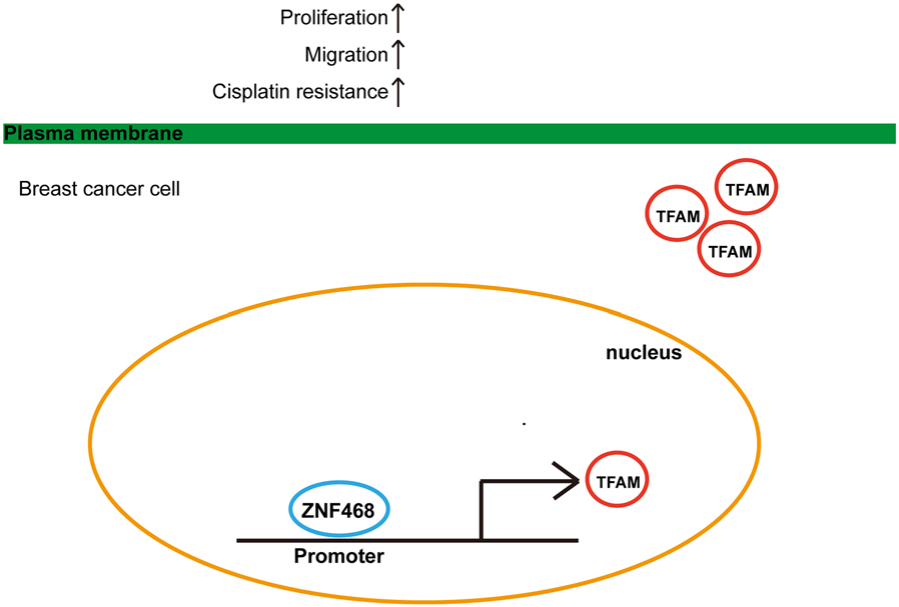
A schematic diagram of ZNF468/TFAM axis in promoting breast cancer cell proliferation, migration and cisplatin resistance

Overall, ZNF468 promotes BC cell proliferation and cisplatin resistance through transcriptional activation of TFAM gene (Fig. 6).

## Discussion

There are approximately 1600 transcription factors (TFs) expressed in eukaryotes, including Kruppel-like family, specificity protein (SP), zinc finger proteins (ZNFs) and etc. [14, 15]. Normal expression of TFs exhibits essential functions on cellular metabolism, proliferation, differentiation, survival and death [16]. Dysregulation of TFs is involved in the development of cancers [17]. To our knowledge, the significance of ZNFs in carcinogenesis is beginning understood. For example, ZNF704 acts as an oncogene in BC [18], chondrosarcoma [19], and uveal melanoma [20] by regulating circadian rhythm and AKT/mTOR signaling. Overexpression of ZNF488 contributes to the malignant progression of nasopharyngeal carcinoma [21] and cervical cancer [22] through activating Wnt and MEK/ERK signaling pathway. In this study, we demonstrated that ZNF468 was overexpressed in BC tissues and cells. High expression of ZNF468 predicted the poor prognosis of BC patients. Up-regulation of ZNF468 contributed to the growth, migration and cisplatin resistance of BC cells.

Mitochondrial transcription factor A (TFAM) is a nuclear-encoded transcription factor that plays an important role in mtDNA replication and damage repair [23]. TFAM is closely associated with the tumor suppressor P53 signaling. Knockdown of TFAM promotes the binding between p53 and E3 ubiquitin protein ligase MDM2, thereby inhibiting p53 expression [24]. TFAM is also involved in p53-mediated apoptosis via direct interacting with p53 [25]. The function of TFAM in carcinogenesis is controversial in different cancer types. While down-regulation of TFAM suppressed the proliferation of HepG2 and U-2 OS cells [26], loss of TFAM facilitates the malignancy of head and neck cancer [27]. Consistent with the promoting function of TFAM in HepG2 and U-2 OS cells, we previously found that TFAM served as an oncogenic protein in BC. However, the upstream regulator of TFAM in BC remains to be determined. In this study, we showed that ZNF468 positively regulated the transcription activity and the protein expression of TFAM gene in BC cells. Mechanistically, ZNF468 interacted with the promoter sequence of TFAM gene, causing the enhanced transcription activity. However, the precise binding area of TFAM promoter was not explored and should be identified in the follow up study. In BC patients, there was a positive correlation between ZNF468 and TFAM. The content of mtDNA, which is regulated by TFAM, was potentiated by ZNF468 overexpression and was suppressed by its knockdown. Importantly, we demonstrated that TFAM up-regulation was critical for the accelerated proliferation and migration ability of BC cells triggered by ZNF468. These results suggest that ZNF468 ectopic expression led to dysfunction of mitochondria and enhanced cell growth capacity via up-regulating TFAM.

Dysregulation of TFAM not only contributes to the progression of malignant tumors, but also regulates the sensitivity of cancer cells to chemotherapeutic drugs, including cisplatin. Down-regulation of TFAM promoted the effectiveness of NSCLC cells to the treatment of cisplatin [28]. In ER positive BC cells, cisplatin resistance was acquired by TFAM overexpression [13]. However, another study showed that knockdown of TFAM attenuated cisplatin-induced cell death in ovarian cancer cells [29]. The results imply that the significance of TFAM in cisplatin effectiveness maybe different in various cancer types. Although we have shown that ZNF468 up-regulation of TFAM is critical for BC cell growth, the involvement of TFAM in cisplatin resistance induced by ZNF468 overexpression needs further validated. As demonstrated by CCK8 and colony formation assays, TFAM knockdown by siRNAs in ZNF468 overexpressed MCF-7 cells significantly restored the sensitivity of cisplatin. Because silence of ZNF468 gene would not be last for 10 days by siRNAs transfection, our study suggested that transient downregulation of ZNF468 could obviously promoted the cytotoxicity of cisplatin, revealing the important function of ZNF468 in BC cell growth and chemotherapy. Nevertherless, stable cell lines with ZNF468 knockdown or overexpression should be constructed in the follow up study. By contrast, TFAM overexpression reduced the cisplatin cytotoxicity in ZNF468 silenced MDA-MB-231 cells. Thus, we confirm that ZNF468 confers cisplatin resistance via up-regulation of TFAM.

## Conclusion

In conclusion, we provided the evidence that ZNF468 contributed to the proliferation and migration of BC cells. Overexpression of ZNF468 alleviated cisplatin effectiveness. Mechanistically, ZNF468 activates the expression of TFAM at transcriptional level. There was a positive correlation between ZNF468 and TFAM in BC patients. Importantly, ZNF468 exerted the tumor-promoting function via up-regulation of TFAM. Our study highlights the potential of ZNF468/TFAM axis as the diagnostic and therapeutic target for BC patients.

## Methods

### The Cancer Genome Atlas (TCGA) analysis and BC specimens

The clinical relevance of ZNF468 and TFAM was analyzed in BC based on the public database, including UALCAN, GEPIA and The Cancer Genome Atlas (TCGA). In brief, the expression of ZNF468 in pan-cancers was analyzed using TCGA data from the UALCAN database (https://ualcan.path.uab.edu/analysis.html). The expression of ZNF468 and TFAM, their correlation, as well as their relationship with BC patients’ overall survival, were analyzed from the GEPIA database (http://gepia.cancer-pku.cn/), which included the data not only from TCGA. A total of 1085 tumor samples and 291 normal tissues were included. For overall survival analysis, the patients were divided into ZNF468 high (n = 212) and low (n = 213) expression group. The results presented in Additional file 1: Fig. S1 were analyzed by using the data from TCGA.

We also collected a total of 12 cancer and normal samples from BC patients who received operation at GuiHang Guiyang Hospital. Each patient signed the informed consent before surgery. This study was approved by the Clinical Research Ethics Committee of GuiHang Guiyang Hospital. The tissues were subjected to immunoblotting analysis of ZNF468.

### Cell culture

Human breast immortalized cell line MCF-10A and BC cell lines MCF-7, MDA-MB-231 and MDA-MB-468 were provided by ATCC (Manassas, VA, USA) or Procell Bio (Wuhan, China). The culture medium was listed as follow: MCF-10A (DMEM:F12 = 1:1, PM150312, Procell), MCF-7 (MEM, PM150410, Procell), MDA-MB-231 and MDA-MB-468 (Leibovitz's L-15, PM151010, Procell). The medium was supplied with 10% of fetal bovine serum (FBS, FND500, ExCell Bio, Suzhou, China) and 1% Penicillin–Streptomycin Solution (PB180120, Procell). The cells were kept in a cell incubator with 37 °C and 5% CO_2_.

### Overexpression and knockdown assay

Knockdown of ZNF468 or TFAM was carried out by transfecting the cells with 20 μM siRNAs using 4 μL RNAiMAX (13,778,030, Invitrogen, Carlsbad, CA, USA), according to the protocols provided by the manufacturer. The cells were collected for other experiments 48 h after transfection. The siRNAs were synthesized from Hippobio (Huzhou, China). siCtrl, 5′-UUCUCCGAACGUGUCACGU-3′; siZNF468#1, 5′-GAAGAAUGUUGCAAAGUUU-3′; siZNF468#2, 5′-GCUUCGAGUUUCAGUGGAA-3′; siTFAM, 5′-GUUGUCCAAAGAAACCUGU-3′.

Overexpression of ZNF468 or TFAM was conducted by transfecting the cells with 5 μg overexpression plasmids containing the coding sequence of indicated gene by using 2 μL VigoFect (T001, Vigorous Bio, Beijing, China). The cells transfected empty plasmids represented the Ctrl group. The cells were subjected for other experiments 48 h after transfection.

### Western blot

The protein samples from BC and normal tissues and cells were extracted using RIPA lysis (P0013C, Beyotime Biotechnology, Shanghai, China). Western blot assay was performed according to the protocols as described previously [30]. Primary antibody against ZNF468 was purchased from Invitrogen. Antibodies against TFAM (#8076, 1:1000) and GAPDH (#2118, 1:10,000) were obtained from Cell Signaling Technology (Danvers, MA, USA). Secondary antibodies were from Proteintech (SA00001-2, 1:10,000; Chicago, IL, USA).

### Real-time quantitative PCR (RT-qPCR)

The RNA was extracted from BC cells transfected with ZNF468 overexpression plasmids or siRNAs using Trizol (15,596,026, Invitrogen), followed by concentration detection on nanodrop and reverse transcription assay with M-MLV reverse transcriptase (M1705, Promega, Madison, WI, USA). Detection of relative cDNA level was conducted on a Bio-Rad qPCR system, as described previously [30]. Primer sequences were listed: TFAM forward, 5′-ATGGCGTTTCTCCGAAGCAT-3′ and reverse, 5′-ATGGCGTTTCTCCGAAGCAT-3′; GAPDH 5′-TGACTTCAACAGCGACACCCA-3′, and reverse, 5′-CACCCTGTTGCTGTAGCCAAA-3.

### Detection of mtDNA

Total DNA was extracted from the cells with the QIAquick Nucleotide Removal Kit (28,304, QIAGEN). Copy number of mtDNA was measured by using the Human Mitochondrial DNA Copy Number Assay Kit (Detroit R&D, MCN1), according to the manufacturer’s instructions and the protocols described previously [31].

### Cell Counting Kit-8 (CCK-8)

The effect of ZNF468 and TFAM on BC cell proliferation and cisplatin sensitivity was detected by CCK-8 (C0039, Beyotime Biotechnology). Briefly, the cells transfected with overexpression plasmids or siRNAs were seeded in 96-well plates, treated with or without different concentrations of cisplatin. One to three days later, 10 μL CCK-8 solution was incubated with the culture medium at 37 °C for 2 h. Cell viability was detected by measuring the OD450 value.

### Colony formation assay

A total of 500 Ctrl and ZNF468 overexpressing MCF-7 cells, and 1000 siCtrl, siZNF468#1 and siZNF468#2 MDA-MB-231 cells were seeded into 6-well plates. The cells were treated with or without 10 μM cisplatin for 7–10 days. When cell colonies formed, the plates were mildly washed by PBS, fixed by methanol for half an hour, and stained by crystal violet. Lastly, the plates were washed by clean water and the colonies were photographed by a camera.

### Transwell analysis of cell migration

A total of 60,000 MDA-MB-231 cells (siCtrl, siZNF468#1, siZNF468#2 and siZNF468#1+TFAM) and 120,000 MCF-7 cells (Ctrl, ZNF468 and ZNF468+siTFAM) in 200 μL FBS-free culture medium were seeded in the upper surface of the transwell chamber. The lower surface was covered onto a total of 500 μL complete medium in 24-well plates. One day later, migrated cells on the lower surface was subjected to methanol fixation and crystal violet staining. Cells attached on the upper surface were removed by the cotton ball. Migrated cells were photographed under the microscope.

### Apoptosis detection

For apoptosis detection, siCtrl, siZNF468#1 and siZNF468#2 MDA-MB-231 cells were harvested by EDTA-free trypsin. After staining with 3 μL PI and 5 μL Annexin V-APC (P-CA-207, Procell), the apoptosis was detected on the flow cytometry system (Beckman).

### Dual luciferase reporter assay

The coding sequence of ZNF468 was cloned into pCDNA3.1 plasmids (Addgene). The promoter sequence of TFAM gene was cloned into pGL3.basic plasmids (Addgene). MCF-7 cells were transfected with empty control and ZNF468 overexpressing plasmids. MDA-MB-231 cells were transfected with siRNAs against negative control and ZNF468. Meanwhile, the cells were transfected with pGL3.basic plasmids containing the promoter of TFAM gene and TK plasmids. Relative dual luciferase activity was determined by using the assay kit from Promega (E1960), according to the manufacturers’ protocols.

### Chromatin immunoprecipitation-qPCR

The coding sequence of ZNF468 with a Flag was cloned into pCDNA3.1 vectors. After transfecting MCF-7 cells with pCDNA3.1-ZNF468/Flag plasmids, the cells were subjected to ChIP assay with IgG and Flag antibody using SimpleChIP enzymatic chromatin IP kit (9005, Cell Signaling), according to the manufacturer’s protocols. Lastly, DNA fragments were quantified by qPCR assay in Chip-IgG and Chip-Flag group. Forward primer, 5′-TGAACAGATGACTTGGAAGG-3′. Reverse primer, 5′-CATTGGCTCTCTGGAAGTAG-3′.

### Statistical analysis

The data were shown as mean ± standard error of mean (SEM) and statistical analysis was conducted on GrpahPad Prism software. Student’s t test was used to analyze the difference between two groups. One-way ANOVA followed by followed by a Tukey’s post hoc test was applied to compare the difference among multiple groups. P value less than 0.05 was considered as statistical significance.

### Supplementary Information

**Additional file 1: Figure S1**. The clinical significance of ZNF468 in different groups of BC patients. (A) Analysis of ZNF468 transcript was conducted in normal tissues and cancer tissues derived from BC patients with different molecular characteristics. *p < 0.05. **p < 0.01. ***p < 0.001. (B–E) Overall survival was analyzed on HER+ (B), LumA (C), LumB (D) and TNBC (E) patients who were divided into ZNF468 high expression and low expression group. The number of the patients and p value were presented in the figure. **Figure S2.** Original WB results.

## Data Availability

All the data generated during this study were included in this published article.
