# Supplementary material for: Zinc finger protein 468 up-regulation of TFAM contributes to the malignant growth and cisplatin resistance of breast cancer cells
Source: Cell Div. 2024 Mar 1;19:8. doi: 10.1186/s13008-024-00113-1 (PMC10908137; doi:10.1186/s13008-024-00113-1)

**Figure S1. The clinical significance of ZNF468 in different groups of BC patients.** (A) Analysis of ZNF468 transcript was conducted in normal tissues and cancer tissues derived from BC patients with different molecular characteristics. *p<0.05. **p<0.01. ***p<0.001. (B-E) Overall survival was analyzed on HER+ (B), LumA (C), LumB (D) and TNBC (E) patients who were divided into ZNF468 high expression and low expression group. The number of the patients and p value were presented in the figure.


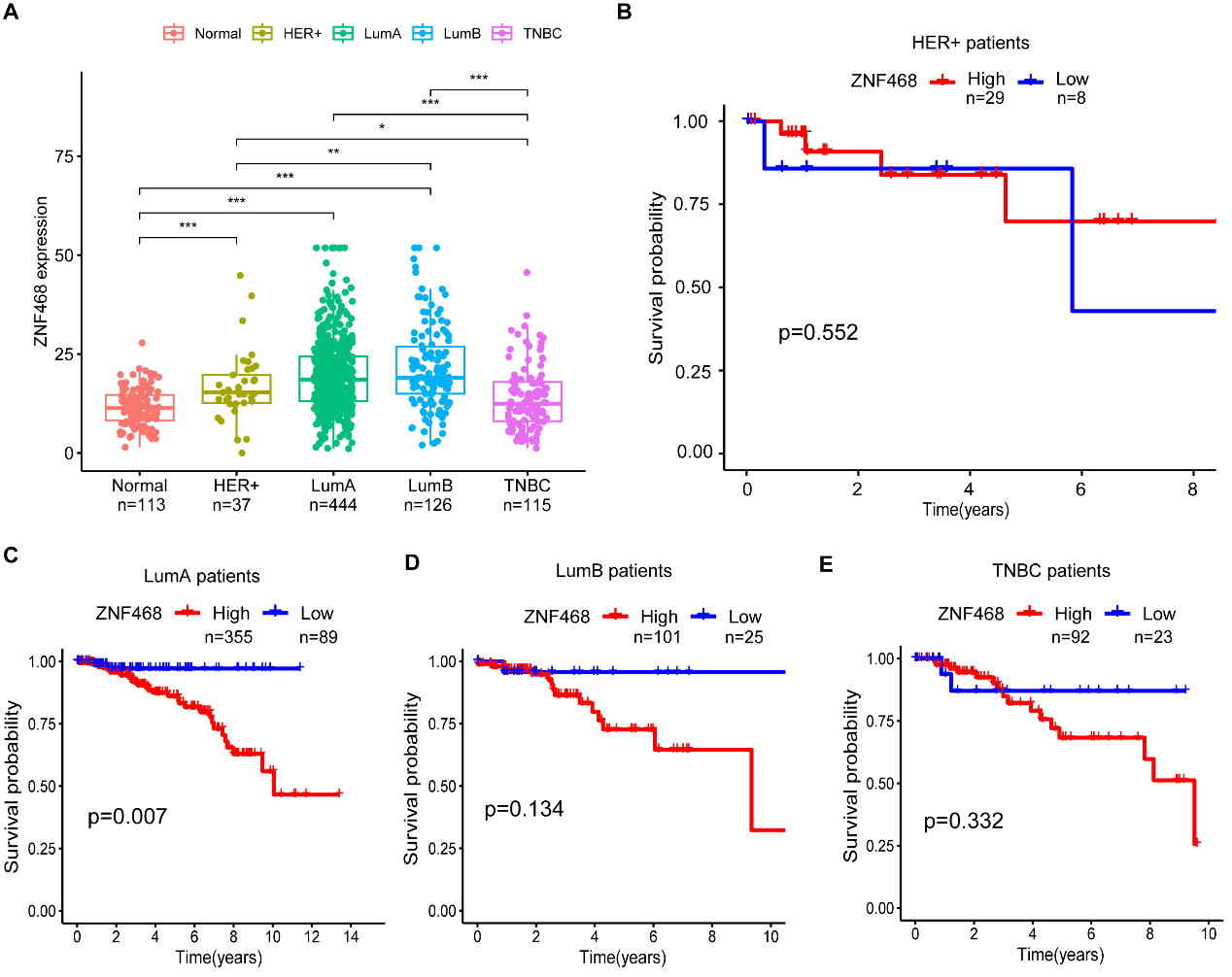


**Figure S2. Original WB results**


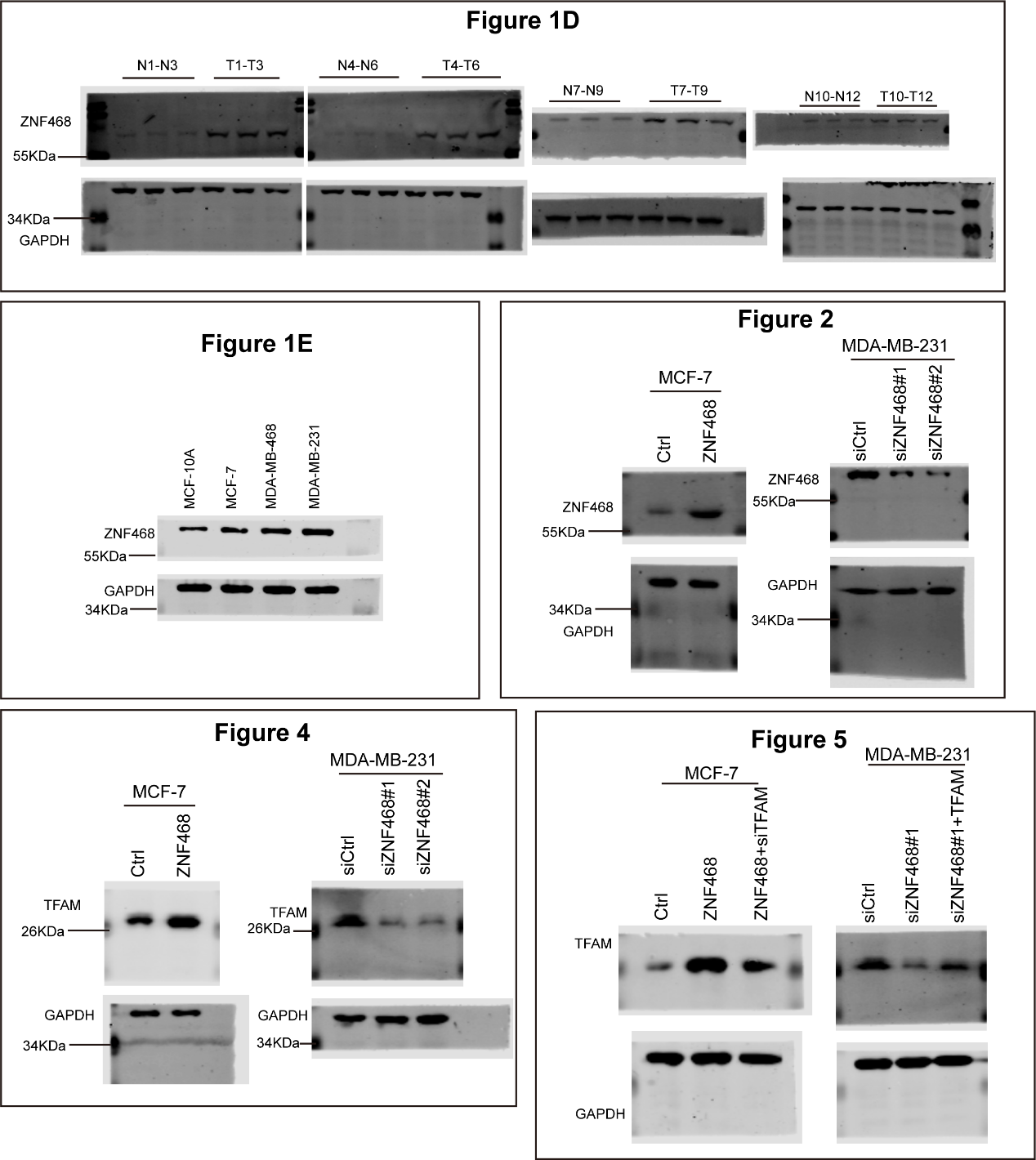

Supplement: Supplementary file 1 — Additional file 1: Figure S1. The clinical significance of ZNF468 in different groups of BC patients. (A) Analysis of ZNF468 transcript was conducted in normal tissues and cancer tissues derived from BC patients with different molecular characteristics. *p < 0.05. **p < 0.01. ***p < 0.001. (B–E) Overall survival was analyzed on HER+ (B), LumA (C), LumB (D) and TNBC (E) patients who were divided into ZNF468 high expression and low expression group. The number of the patients and p value were presented in the figure. Figure S2. Original WB results. [file 13008_2024_113_MOESM1_ESM.docx]
